# Effects of trehalose on NFE2L2, catalase, and superoxide dismutase in the kidney of aged rats

**DOI:** 10.22099/mbrc.2022.42014.1688

**Published:** 2022-03

**Authors:** Yaser Hozhabri, Asie Sadeghi, Mahdieh Nazari-Robati, Faegheh Bahri, Fouzieh Salimi, Moslem Abolhassani, Abbas Mohammadi

**Affiliations:** 1 Neuroscience Research Center, Institute of Neuropharmacology, Kerman University of Medical Sciences, Kerman, Iran; 2 Department of Clinical Biochemistry, Faculty of Medicine, Kerman University of Medical Sciences, Kerman, Iran

**Keywords:** Trehalose, Oxidative stress, NFE2L2, SOD, CAT

## Abstract

Aging is associated with an increase in oxidative stress, which damages organs such as the kidney. Trehalose has abundant beneficial activities including antioxidative effects. This study aimed to investigate the effects of trehalose on several antioxidant parameters of the aged kidney. Wistar rats were divided into three groups: young (4 months), aged (24 months), and aged-trehalose. The third group was treated with 2% trehalose for one month. The expression of target genes and enzyme activities in the kidney of the animals were evaluated by quantitative polymerase chain reaction (qPCR) and enzyme colorimetric procedures, respectively. Protein levels of NFE2L2 showed a 50% reduction in aged rats compared to young rats (P<0.001), which was restored by trehalose intervention. In addition, the activity and mRNA levels of catalase (CAT) increased in aged rats while treatment with trehalose reversed this trend. On the other hand, superoxide dismutase (SOD) activity was reduced in the kidneys of aged rats but was not affected by trehalose intervention .It is concluded that trehalose supplementation alleviates the antioxidant system impairments in the kidneys of aged rats. However, further investigations are needed to thoroughly describe the antioxidative impacts of trehalose on the kidneys during aging.

## INTRODUCTION

Aging is a dynamic and continuous process that occurs over time at the different levels of organs, tissues, cells, and molecules, causing the gradual loss of tissues as well as the deterioration of the function of organs over time. Although the mechanism of aging is complex and unknown, a number of studies have reported the key role of oxidative stress in age-related disorders [1, 2]. With the increase of age, the balance between reactive oxygen species (ROS) and antioxidants is disturbed, leading to the oxidative damage of lipids, proteins, and DNA, and ultimately decreased immune function, increased inflammation, apoptosis, and tissue damage [1]. It should be noted that kidneys are more prone to the damage caused by oxidative stress due to the abundance of long-chain unsaturated acids found in the structure of their lipids [3]. Hallmarks of aging in kidneys include decreased renal mass, low glomerular filtration rate (GFR), tubular atrophy, glomerular basement membrane thickening, and glomerulosclerosis [4].

As age increases, the expression of nuclear factor erythroid 2-related factor 2 (NFE2L2), which is a regulator of oxidative stress, decreases and cells become more sensitive to oxidative stress; thus, the aging phenotype becomes more apparent [5]. Under normal homeostatic conditions, the transcription factor NFE2L2 is inhibited by Kelch-like ECH-associated protein 1 (Keap1) in the cytoplasm. However, upon exposure to ROS, NFE2L2 is released from the Keap1 inhibitor and transferred to the nucleus, where it binds to the antioxidant response elements (AREs) [6] and induces the production of endogenous antioxidant enzymes including superoxide dismutase (SOD), catalase (CAT), peroxidase, co-oxygenase, and NADPH quinone oxidoreductase 1 (NQO1), as well as the glutathione system [7]. 

Trehalose, which is a non-reducing disaccharide, is produced in various organisms such as plants, bacteria, yeasts, insects, and invertebrates. This disaccharide protects the cell against stressors such as cold, heat, drought, and ROS [8]. It has been suggested that trehalose can be used as a treatment strategy for age-related diseases [9]. The effects of trehalose against ROS, which lead to the subsequent reduction of lipid peroxidation [10] and protein damage [11] have also been reported in various studies. Mizunoe et al., suggested that trehalose protected against oxidative stress by enhancing the expression of antioxidant genes via regulating the Keap1–NFE2L2 signaling pathway [12]. In addition, Liu et al. showed that trehalose improved ischemic-reperfusion impairment by increasing autophagy mediators and inhibiting inflammatory responses, apoptosis, and oxidative stress [13]. 

To determine the oxidative damage of kidneys in aging and the antioxidant characteristics of trehalose, we investigated the effect of trehalose on several components of the antioxidant system in the kidneys of aged rats. For this purpose, we measured the protein levels and gene expression of NFE2L2 and examined the expression and activity of SOD and CAT in the studied groups.

## MATERIALS AND METHODS

**Animals: **In this study, 24 male Wistar rats including 16 aged male rats (24 months old) with a weight of about 350 to 400 g, and 8 young male rats (4 months old) with a weight of about 200 to 250 g were used. The animals were kept in a controlled environment with a steady temperature of 22°C under 12-h light/dark cycles. After one week of acclimation, aged rats were divided into two groups including the aged control group and the aged trehalose-treated group. The aged trehalose-treated group consumed 2% trehalose in water for one month whereas the other groups received tap water. All animals were fed a standard laboratory diet. After a one-month intervention, the animals were sacrificed and their kidneys were removed and used for the next experiments. The experimental protocol was approved by the ethics committee of Kerman University of Medical Sciences (1399.098.).

**RNA extraction, cDNA synthesis, and real-time polymerase chain reaction (PCR): **RNA was manually extracted from the kidney tissue using Trizol (Gene All, Korea). Briefly, the kidney tissue was homogenized in the Trizol reagent with a homogenizer followed by treatment with chloroform. In this step, RNA was extracted to the aqueous phase. The concentration and purity of the extracted RNA were measured by a Nanodrop spectrophotometer. cDNA was synthesized using a cDNA synthesis kit (Yekta Tajhiz, Tehran, Iran). Then genes of interest were amplified using the master mix SYBR green kit (Amplicon, Denmark) and specific primers (Table 1) [12, 14-17] in a Mic qPCR Cycler. The selected primers in this study were blasted by Primer Blast Software. In addition, at the end of the qPCR run, the melt curve for each gene was created and subsequently analyzed, indicating that the implication with each primer pair was specific.

 The temperature program of PCR included an initial denaturation step at 95°C for 15 min followed by 40 cycles. Each cycle consisted of a denaturation step at 95°C for 25 s, a primer annealing step at 62°C for 30 s, and an elongation step at 72°C for 30 s. The differences in the expression of genes of interest between groups were then determined using the 2^-∆∆ct^ method in which glyceraldehyde-3-phosphate dehydrogenase (GAPDH) was used as an internal control gene [18].

**Table 1:** Sequences of primers used for quantitative PCR

**Gene**	**Forward Primer (5′→3′)**	**Reverse Primer (5′→3′)**	**Product size(bp)**	**Refrence**
SOD1	GCAGAAGGCAAGCGGTGAAC	CGGCCAATGATGGAATGCTC	282	[17]
*SOD2*	CCCAAAGGAGAGTTGCTGGAG	CGACCTTGCTCCTTATTGAAGC	137	[12]
*NFE2L2*	CACATCCAGACAGACACCAGT	CTACAAATGGGAATGTCTCTGC	121	[14]
Catalase	TTCTACACTGAAGATGGTAACTG	GAAAGTAACCTGATGGAGAGAC	189	[15]
*GAPDH*	AGGTTGTCTCCTGTGACTTC	CTGTTGCTGTAGCCATATTC	130	[16]

**Measurement of NFE2L2 protein: **First, the kidney tissue samples were homogenized in cold phosphate buffer saline. An anti-protease cocktail was applied to preserve proteins during sample preparation. After centrifugation, the supernatant was isolated and the NFE2L2 protein was measured using the enzyme-linked immunosorbent assay (ELISA) kit (Zell Bio, Germany). Briefly, 40 μL of the sample, 10 μL of NFE2L2 antibody, and 50 μL of streptavidin-HRP were added to the wells and incubated at 37°C for 60 min. The wells were washed with diluted wash buffer and then the chromogen solution was loaded and incubated at 37°C for color development. Finally, the reaction was stopped and the optical density was measured at 450 nm.

**Measurement of SOD activity: **SOD activity assay was carried out using pyrogallol. Pyrogallol is oxidized in the presence of superoxide radicals, and this reaction is inhibited by SOD. Briefly, 100 µL of EDTA-tris buffer and 10 µL of the sample were added to each well of a 96-well plate and the absorbance was read at 420 nm. In the next step, 100 µL of the pyrogallol solution was loaded into the wells and the absorbance was reread at 420 nm. Finally, the enzyme activity percentage was calculated using the obtained absorbances.

**Measurement of CAT activity: **In order to measure CAT activity in the kidney tissue homogenates, a colorimetric method applying a K_2_Cr_2_O_7_/acetic acid reagent was used. Briefly, 66 µL of H_2_O_2_ solution (0.1% H_2_O_2_ in phosphate buffer) and 60 µL of the sample were poured into a microtube and incubated at 37°C for 3 min. Then, the K_2_Cr_2_O_7_/acetic acid reagent was added to the solution and incubated at 100°C for 10 min. The absorbance of the samples was read at a wavelength of 570 nm.

**Statistical analysis** **: **IBM SPSS version 16 was used to analyze the data. Significant variations of means among the groups were determined by one-way ANOVA followed by Tukey's post hoc test (P≤0.05). All tests were performed at least three times. The results were reported as mean ± SEM.

## RESULTS

**Figure 1:**
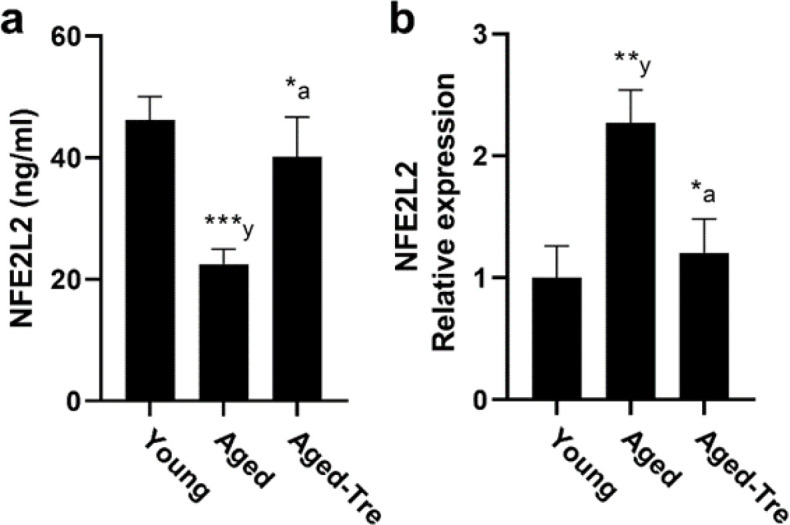
The effects of trehalose on the protein and mRNA levels of NFE2L2 in the kidneys of aged rats. (a) NFE2L2 protein levels, (b) NFE2L2 mRNA levels. Aged-Tre: Aged rats that received 2% trehalose in water for 1 month; y: Compared to young rats; a: Compared to aged rats. *p<0.05, **p<0.01, ***p<0.001

As shown in Figure 1, the protein levels of NFE2L2 were lower in the kidneys of aged rats compared to the young group (22.53 and 46.34 ng/mL, respectively; P<0.001). However, the treatment of aged rats with trehalose restored the NFE2L2 protein levels (P=0.03). The mRNA expression of *NFE2L2* showed a 2.27-fold increase in the kidneys of aged rats compared to young rats (P=0.005). However, it was decreased in aged animals treated with trehalose compared with the aged control rats (P= 0.016). 

**Figure 2:**
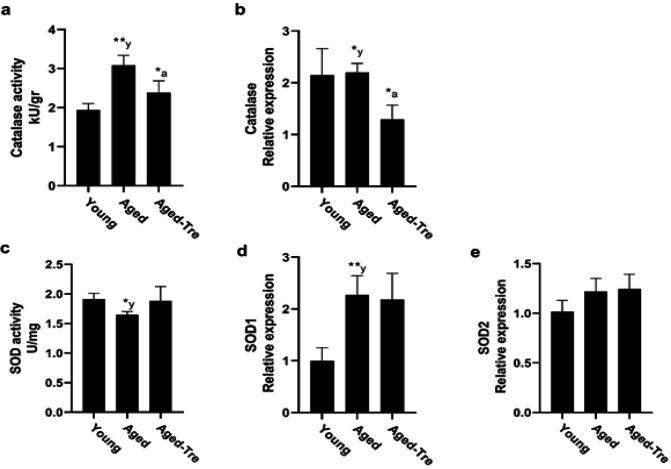
The effects of trehalose on the mRNA expression and activity of antioxidant enzymes in the kidneys of aged rats. (a) Catalase activity, (b) Catalase mRNA levels, (c) SOD activity, (d) SOD1, and (c) SOD2 mRNA levels. Aged-Tre: Aged rats that received 2% trehalose in water for 1 month; y: Compared to young rats; a: Compared to aged rats. *p<0.05, **p<0.01, ***p<0.001

 Compared with the young group, a significantly higher level of CAT activity was detected in the kidney tissue of the aged group (P<0.01), whereas this activity was significantly decreased in aged rats treated with trehalose compared to the aged control group (P<0.05) (Figure 2a). In contrast with the CAT activity, SOD activity decreased in the aged group compared with the young group (P<0.05). There were no statistical differences in SOD activity between the aged control group and aged rats treated with trehalose (Figure 2c). The mRNA levels of CAT and SOD1 were significantly upregulated in the kidneys of aged rats (P=0.01 and P=0.005, respectively) while no significant change was observed in the mRNA levels of SOD2 (Figures 2b and 2d). The mRNA levels of CAT were decreased in aged rats after treatment with trehalose (P=0.036). However, trehalose did not change the mRNA levels of SOD1 and SOD2 (Figures 2d and 2e).

## DISCUSSION

Oxidative stress is a harmful phenomenon, which is augmented with aging and contributes to the impaired function of various organs such as kidneys during aging. Trehalose has the potential to improve the function of an aging body because of its antioxidant properties. Therefore, in this study, the effects of trehalose on several components of the antioxidant system in the aged kidney tissue were investigated. The present study also revealed that the treatment of aged rats with trehalose ameliorated adverse alterations in the kidneys. 

NFE2L2 transcription factor is activated in response to oxidative stress. Upon its binding to ARE in the promoter of target genes, it regulates the redox status of cells. Several studies have indicated that NFE2L2 signaling was impaired in tissues such as the skeletal muscle [19] and liver [20] with the increase of age. Similarly, in the present study, the protein levels of NFE2L2 were found to be decreased in the kidneys of aged rats while its mRNA levels were upregulated. No similar patterns of mRNA and protein levels of NRF2 were observed in hepatocytes and cochleae during aging, and NRF2 mRNA exhibited no significant change despite a decline in its protein levels [21, 22]. These inconsistencies in the results are partly explained by the variation in the exact age of the studied animals. However, it appears that as age increases and oxidative stress is elevated, NRF2 transcription is upregulated as a compensatory response but it fails to translate to proteins, suggesting that mRNA translation or post-translational modifications of NFE2L2 may be affected in early aging rather than transcription.

This study also showed significant alterations in both mRNA and protein levels of antioxidant enzymes SOD and CAT in the kidney of aged rats in comparison with young rats. Our data demonstrated that the mRNA levels and activity of CAT increased in aged rats while the SOD activity was reduced in these rats. However, SOD mRNA, especially SOD1, increased in aged rats compared to young rats, unlike what was observed for SOD activity. Consistent with our results, GU et al. reported that the expression of SOD mRNA in the liver of 340-day-old laying hens was higher than that of 195-day-old and 525-day-old groups [23]. SOD1 was shown to be upregulated in the human myocardia tissue of 55-year-old individuals compared to 22- and 72-year-old individuals [24]. These results suggested that with the increase of age, in early aging, antioxidant genes including SOD are upregulated but fail to produce functional proteins. 

Since a decline in SOD activity leads to an increased ROS, particularly superoxide radicals, it appears that the elevation of CAT activity may be a compensatory response. In agreement with the results of the current study, an increase in CAT activity and a decrease in SOD activity were observed in patients with multiple sclerosis, which is an age-related disease [25]. Collectively, it can be concluded that the levels of antioxidant enzymes elevate as a compensatory response to increased oxidative stress during early aging. However, the accumulation of oxidative species over time subsequently results in an exhausted antioxidant system. This phenomenon can be considered a reason for the decline in most of the components of the antioxidant system during aging [26, 27]. 

Trehalose is a potent antioxidant with known beneficial effects. The protective effects of trehalose against oxidative stress were observed in various tissues and cells including the liver [28], brain [29], spleen [30], and peripheral blood mononuclear cells (PBMCs) [31]. However, there is no report on the effect of trehalose on the oxidative stress associated with aging in the kidneys. This is the first study to show that trehalose is capable of restoring the alterations of the antioxidant defense system due to aging in the kidney. Trehalose enhanced NFE2L2 protein level and decreased CAT activity while it had no effect on SOD. Considering the observed decrement in antioxidant enzyme activity following trehalose treatment, it is plausible that trehalose directly interacts with ROS, thereby neutralizing them. However, some studies reported that trehalose promoted the activities of antioxidant enzymes [32]. Regarding the molecular mechanisms that underlie the antioxidative effects of trehalose, some studies have indicated that this molecule exerts its effects partly through the NFE2L2 pathway. Trehalose is shown to upregulate NFE2L2 expression in the liver and brain of aged animals [1]. It was also demonstrated that trehalose increases NFE2L2 activity by stimulating its nuclear translocation [1, 12]. In this study, the total protein level of NFE2L2 increased by trehalose intervention; however, the subcellular localization of NFE2L2 was not investigated, which is one of the main limitations of the present work. Another limitation is that no other antioxidant enzymes were measured. In the present study, it was found that NFE2L2 was not positively associated with the mRNA expression of CAT and SOD activity, indicating that other transcription factors are possibly responsible for the higher expression of SOD1 and CAT. In addition to NFE2L2, other transcription factors such as NF-κB, AP-1, AP-2, and Sp1 are demonstrated to control the transcription of *SOD* genes [33] and CAT expression is regulated by PPARγ and p53 [34]. 

In summary, it is concluded that trehalose supplementation alleviates the impairments of the antioxidant system in the kidneys of aged rats. However, further investigations are needed to thoroughly describe the antioxidant effects of trehalose on the kidney during aging. 

## Conflict of Interest

The authors declare no potential conflict of interest.
